# Atrogin1-induced loss of aquaporin 4 in myocytes leads to skeletal muscle atrophy

**DOI:** 10.1038/s41598-020-71167-8

**Published:** 2020-08-25

**Authors:** Seok Won Chung, Ja-Yeon Kim, Jong Pil Yoon, Dong Won Suh, Woo Jin Yeo, Yong-Soo Lee

**Affiliations:** 1 Department of Orthopedic Surgery, Research Institute of Medical Science, Konkuk University School of Medicine, Seoul, Republic of Korea; 2 Department of Orthopedic Surgery, School of Medicine, Kyungpook National University, Daegu, Republic of Korea; 3 Joint Center, Barunsesang Hospital, #75-5, Yatap-ro, Seongnam-si, Gyeonggi-do 13497 Republic of Korea

**Keywords:** Molecular biology, Pathogenesis

## Abstract

The water channel aquaporin 4 (AQP4) regulates the flux of water across the cell membrane, maintaining cellular homeostasis. Since AQP4 is enriched in the sarcolemma of skeletal muscle, a functional defect in AQP4 may cause skeletal muscle dysfunction. To investigate a novel mechanism underlying skeletal muscle atrophy, we examined AQP4 expression and its regulation in muscle using the rotator cuff tear (RCT) model. Human and mouse AQP4 expression was significantly decreased in atrophied muscle resulting from RCT. The size and the number of myotubes were reduced following AQP4 knockdown. Atrogin 1-mediated ubiquitination of AQP4 was verified with an ubiquitination assay after immunoprecipitation of AQP4 with an anti-AQP4 antibody. In this study, we identified high mobility group box 1 (HMGB1) as a potent upstream regulator of atrogin 1 expression. Atrogin 1 expression was increased by recombinant mouse HMGB1 protein, and the HMGB1-induced atrogin 1 expression was mediated via NF-κB signaling. Our study suggests that loss of AQP4 appears to be involved in myocyte shrinkage after RCT, and its degradation is mediated by atrogin 1-dependent ubiquitination. HMGB1, in its function as a signaling molecule upstream of the ubiquitin ligase atrogin 1, was found to be a novel regulator of muscle atrophy.

## Introduction

Aquaporins (AQPs) are a family of transmembrane proteins that transport water and, in some cases, glycerol and urea across cell membranes^1,2^. These proteins are involved in a variety of cellular functions associated with water movement, such as urinary concentration, brain function, glandular secretion, skin hydration, male fertility, and body fluid homeostasis. Therefore, defects in AQP function have been implicated in various diseases and pathological states^3^. AQP4 is the predominant water channel type in skeletal muscle, particularly in the sarcolemma of fast-twitch skeletal muscle fibers^4^. Skeletal muscle AQP4 expression is significantly decreased in the *mdx* mouse model of Duchenne muscular dystrophy^4,5^. In addition, it has been reported that reduced AQP4 expression is often associated with markedly reduced α1-syntrophin levels^6,7^. A previous study has demonstrated the physiological role of AQP4 in supporting muscle contractile activity and the metabolic changes that occur in fast-twitch skeletal muscle during prolonged exercise^8^. It has also been demonstrated that muscle activity modulates sarcolemmal expression of AQP4, which promotes water exchange between blood and muscle fibers to regulate volume changes during muscle use^9^. Skeletal muscle plays an important role in the maintenance of osmotic equilibrium in the human body, and rapid fluid exchange occurs in this tissue after intense use. Taken together, these findings support the idea that loss of AQP4 may be associated with skeletal muscle dysfunction.

Skeletal muscle atrophy involves progressive degeneration of myocytes leading to reduced muscle mass^10^. Muscle atrophy is triggered by various factors, including aging, cancer, cachexia, injury, inflammation, immobilization, neural inactivity, mechanical unloading, metabolic stress, and elevated glucocorticoid levels^11,12^. It is ultimately derived from an imbalance between anabolic and catabolic processes, with loss of muscle mass when protein degradation exceeds protein synthesis^13,14^. Three major protein degradation pathways are found in eukaryotic cells: the ubiquitin–proteasome system (UPS), the autophagy–lysosome pathway, and apoptosis. With respect to skeletal muscle atrophy, UPS is known to be an important protein degradation mechanism, and the representative muscle-specific E3 ligases muscle atrophy F-box (MAFbx)/atrogin 1 and muscle RING finger 1 (MuRF1) are associated with this system^14,15^. These E3 ligases function by binding and facilitating the ubiquitination of their cognate substrates, which are subsequently degraded by the 26S proteasome. As key markers of skeletal muscle atrophy, mRNA expressions of both *atrogin 1* and *MuRF1* are rapidly upregulated upon onset of a variety of atrophy-inducing conditions and prior to the onset of muscle loss. To date, various regulators of atrogin 1 and MuRF1 expression have been identified and their functions have been investigated^16,17^. However, the molecules and cellular pathways regulating skeletal muscle atrophy remain largely unknown. Moreover, studies concerning the identification of the cellular targets of atrogin 1 and MuRF1 are scarce^18,19^, and this remains an active area of research.

As atrophy-associated genes, expression levels of *atrogin 1* and *MuRF1* are upregulated by muscle unloading, disuse, nerve injury, inflammation, and other metabolic stresses^14^. Among these triggers, the inflammatory response is induced very rapidly, and plays a wide range of roles in skeletal muscle homeostasis through innate immune receptors on myocytes^20^. High mobility group box 1 (HMGB1) is a nuclear protein that exerts divergent effects on cells. In the nucleus, this protein acts as an architectural chromatin-binding factor that bends DNA and promotes protein assembly on specific DNA targets. However, when membrane integrity is lost, as in permeabilized or necrotic cells, nuclear HMGB1 rapidly leaks into the cytoplasm, where it promotes innate and adaptive immune responses and exhibits cytokine activity^21,22^. The inflammatory functions of HMGB1 occur via receptors, including receptor for advanced glycation end products (RAGE) and Toll-like receptors (TLRs)^22^. HMGB1 plays multiple roles in the pathogenesis of inflammatory diseases and mediates immune responses that range from inflammation to tissue repair. However, its contribution to immune responses in muscle during infections or atrophy is not fully understood.

Rotator cuff tear (RCT) is a muscle injury representative of various musculoskeletal complaints, and fatty infiltration and muscle atrophy in this condition are associated with poor clinical outcomes and failed rotator cuff repair^23,24^. Given that RCT, in various animal species, results in pathological changes similar to those seen in humans, including muscle atrophy and fatty infiltration, such models are considered a good approach to understanding a variety of muscular disorders^25,26^. In this study, we hypothesized that a defect in the function of the water-transporting protein, AQP4, may be associated with skeletal muscle dysfunction, such as muscle atrophy. To test this hypothesis, we investigated the expression of AQP4 as well as that of its upstream regulator in our mouse model of RCT.

## Results

### AQP4 expression in human rotator cuff muscles after RCTs

**Figure 1:**
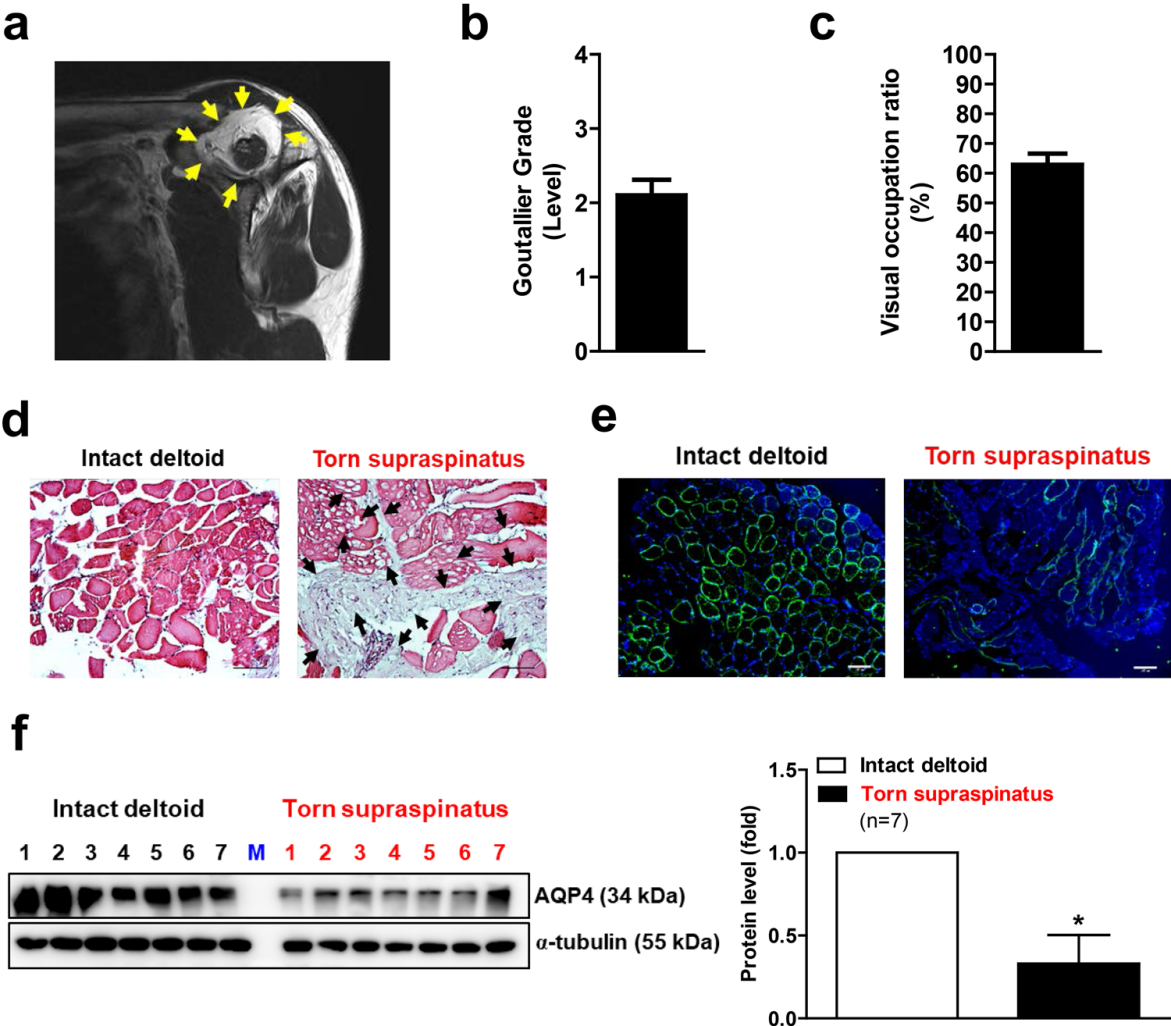
Muscle atrophy and reduced AQP4 expression in the supraspinatus muscles of patients with a rotator cuff tear (RCT) (n = 9). (**a**) Sagittal images of postoperative MRI. Picture is a representative of independent MRI image. Yellow arrows indicate fatty infiltration in supraspinatus muscle of RCT patient. (**b**) The fatty infiltration of supraspinatus muscles of RCT patient was assessed using a Goutallier classification as per the following grading: grade 0, normal; grade 1, some fatty streaks; grade 2, more muscle than fat; grade 3, as much fat as muscle; and grade 4, more fat than muscle. (**c**) Visual occupation ratios were categorized based on a three-point scale: grade 1 involved minimal to mild atrophy of the supraspinatus muscle and an occupation ratio ≥ 60%; grade 2 involved moderate atrophy, 60% > occupation ratio ≥ 40%; and grade 3 represented severe atrophy with an occupation ratio < 40%. Age, sex, Goutallier grade, and visual occupation ratio of each patient is shown in ‘Supplementary Table 1’. (**d**) Histological difference between intact deltoid muscle (as a control) and supraspinatus muscle of RCT patient. Arthroscopically acquired muscle samples from the edge of the torn rotator cuff tendon were used for hematoxylin and eosin (H&E) staining. Black arrows indicate fatty infiltration within the muscle. Pictures are representative of independent tissue samples (n = 9). Scale bar: 100 μm. (**e**) Immunofluorescence (IF) microscopy analyses for AQP4 expression. Pictures are representative of independent tissue samples (n = 9). Scale bar: 100 μm. The negative control (secondary antibody only) for the IF showed no specific signal (data not shown). (**f**) Western blot analyses for AQP4 protein expression between intact deltoid muscle and supraspinatus muscle of patients with RCTs (n = 7). Densitometric analyses of the western blots are shown on the right. Data represent means ± standard errors of the means. *P < 0.05 (**c**).

We first examined the expression levels of human AQP4 in atrophied muscles. For this examination, we evaluated the grade of fatty infiltration and atrophy of rotator cuffs in preoperative MRI in patients with RCTs. The oblique sagittal images of postoperative MRIs revealed remarkable fatty infiltration around the injured area (Fig. 1a). The supraspinatus muscle of a patient with an RCT showed a significant fatty infiltration and atrophy, which were evaluated using a Goutallier classification system^23^ and a visual occupation ratio, respectively. (Fig. 1b,c). The isolated supraspinatus muscle from a RCT patient also revealed abundant ectopic fatty accumulation compared to the intact deltoid muscle (Fig. 1d). Interestingly, the immunofluorescence analysis revealed that the AQP4 protein expression was markedly reduced in the torn supraspinatus muscle compared with intact deltoid muscle (Fig. 1e). Western blot analysis also showed a significant decrease in AQP4 protein expression in the supraspinatus muscle (Fig. 1f), suggesting that AQP4 expression was reduced after RCT in humans.

### RCT-induced muscle atrophy is accompanied by decreased AQP4 protein levels in mice

**Figure 2:**
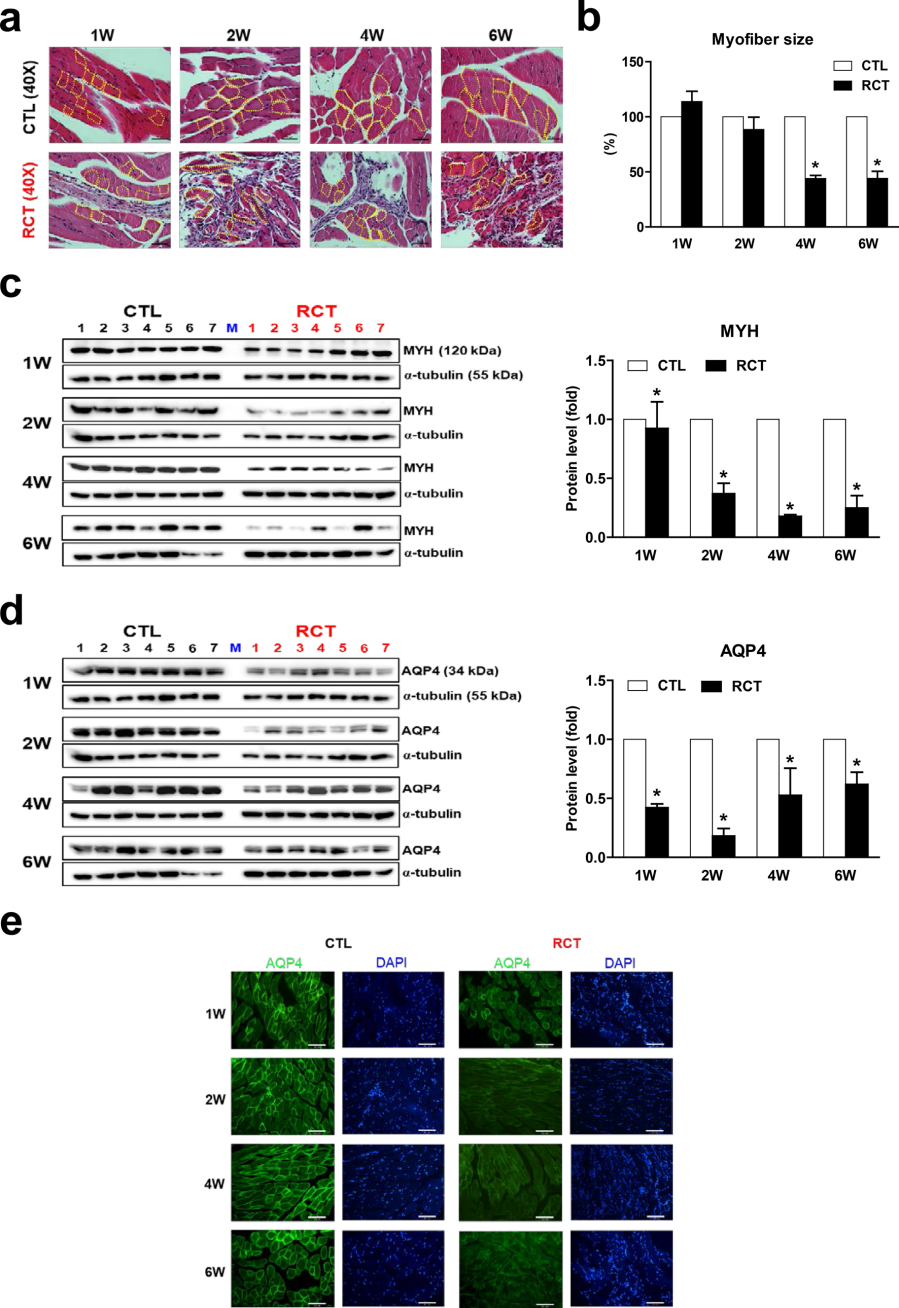
Reduced aquaporin 4 (AQP4) protein levels in atrophied muscle following rotator cuff tear (RCT). (**a**) Reduced myofiber size in muscle injured by RCT. Magnified view of the H&E-stained muscle. Magnification, × 400. Scale bar, 50 μm. The yellow dotted line indicates the muscle myofiber. (**b**) Myofiber size in control (CTL) and RCT muscles. The size of each dotted area was measured using ImageJ software. *P < 0.05. (**c**) Western blot analyses for myosin heavy chain (MYH) protein expression between control and the injured muscle after RCT (n = 7 per group). Densitometric analyses of the western blots are shown in the graphs. Data represents mean ± SEM. *p < 0.05. (**d**) Reduced aquaporin 4 (AQP4) protein levels in atrophied muscle following RCT (n = 7 per group). Densitometric analyses of the western blots are shown on the right. Data represent means ± standard errors of the means. *P < 0.05. (**e**) Immunofluorescence microscopy analyses of muscle cells isolated at different stages after RCT (n = 3 per group). The green signal in each panel represents membranous AQP4 protein and the blue signal represents cell nuclei. Magnification, × 200. Scale bar, 100 μm.

To examine the correlation between muscle atrophy and reduced levels of AQP4, we initially established a mouse RCT model as described previously^27^ (Supplementary Fig. 1a). Histological analysis revealed an acute inflammatory response in the initial stage of RCT (1-week post-surgery). Ectopic fat accumulation was observed 2 weeks after RCT, and muscular fatty infiltration markedly increased in the injured region thereafter (Supplementary Fig. 1b). With respect to this phenomena, the inflammation-associated genes, *Il-1b* and *Il-6*, were markedly upregulated 1 week after RCT, and the adipogenic genes, *Cebpa* and *Pparg*, were significantly upregulated 2 weeks post-RCT, along with the myogenic regulators, *Myod1* and *Myf5* (Supplementary Fig. 1c). Importantly, myofiber size was significantly reduced and myocytes were smaller in the injured muscle than the control tissue, suggesting that RCT caused substantial muscle atrophy (Fig. 2a,b). We also confirmed that the expression of myosin heavy chain (MYH), which is depleted under atrophy conditions, was significantly reduced by RCT (Fig. 2c).

To test whether AQP4 protein levels are negatively affected by RCT, we performed western blot analysis. Interestingly, AQP4 protein expression was significantly decreased 1 week after RCT and dramatically reduced by week 2 (to less than 25% of that in the control group) but restored (to nearly 70% of control group levels) by week 6 (Fig. 2d). Immunofluorescence staining clearly showed the presence of AQP4 on myocyte membranes, and a remarkable reduction of AQP4 protein levels in injured myocytes (Fig. 2e). This diminished protein expression was not caused by its gene expression although there was markedly decreased *Aqp4* mRNA expression 1 week after RCT (Supplementary Fig. 2). Taken together, these results suggested that muscle injury associated with RCT induces a reduction in AQP4 protein expression and that AQP4 loss may be directly related to muscle atrophy.

### Knockdown of AQP4 lead to decrease in muscle cell size and population

**Figure 3:**
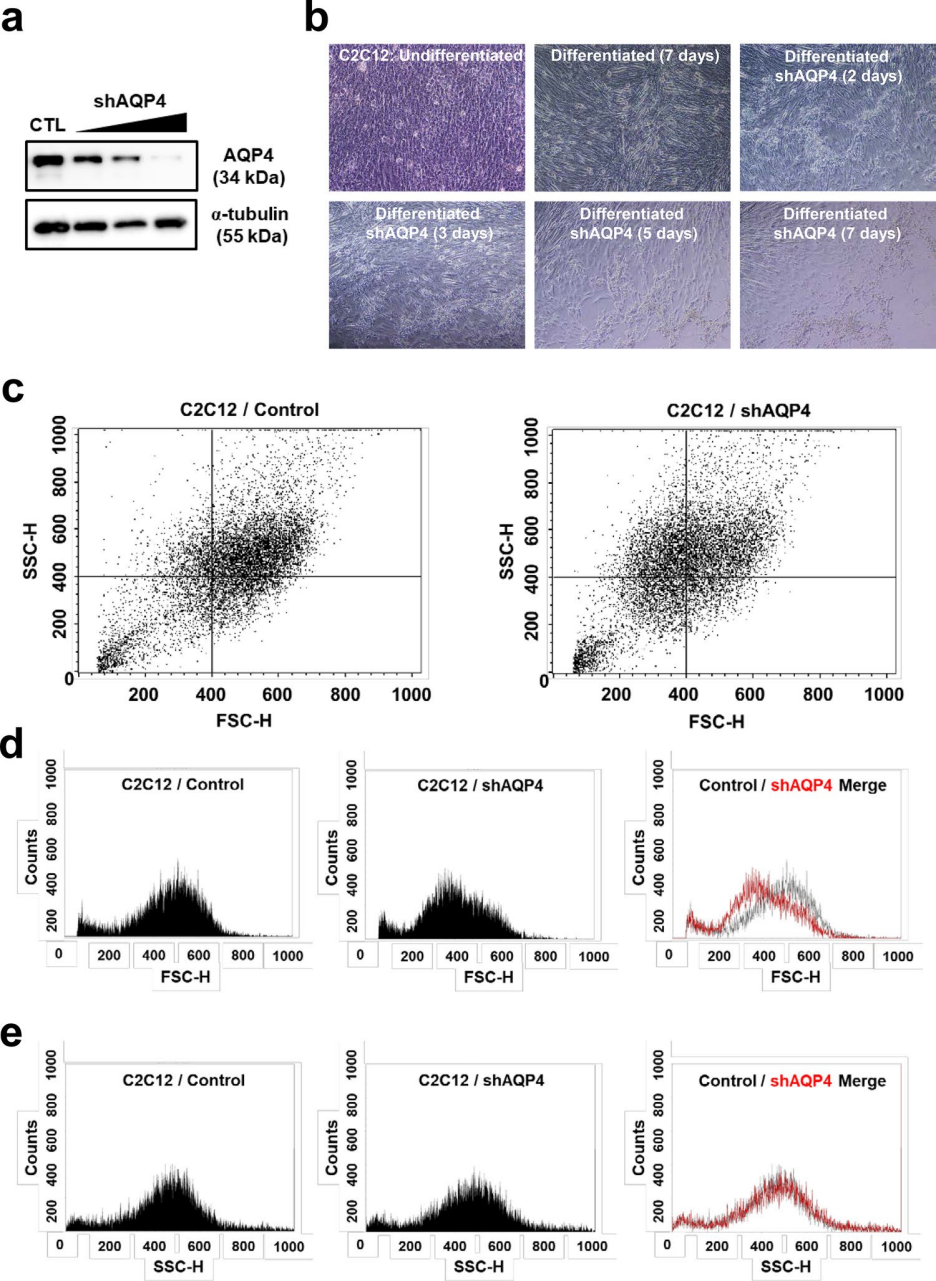
Knock down of AQP4 and cell atrophy. (**a**) AQP4 protein expression in AQP4 shRNA-transfected C2C12 cells. Differentiated C2C12 cells were transfected with a different dose of shAQP4 for 48 h, and the cell lysates were used for western blot analysis for AQP4 protein expression. (**b**) Morphology of AQP4 shRNA-transfected C2C12 cells during differentiation. C2C12 cells were differentiated for 7 days with or without AQP4 shRNA. (**c**) Flow cytometry analysis in AQP4 shRNA-transfected C2C12 cells. Differentiated C2C12 cells were transfected with shAQP4 for 48 h, and the size and granularity (complexity) of cells were determined by forward and side scatter (FSC and SSC) gating, respectively. Forward scatter (FSC) represent the cell size (**d**), and side scatter (SSC) data represent the complexity or granularity of the cell (**e**).

To verify whether loss of AQP4 substantially correlates with muscle atrophy, we knocked down AQP4 by transfecting shRNA against AQP4 in C2C12 cells (Fig. 3a). In the presence of AQP4 shRNA, the differentiated myotubes significantly shrank and the cell population was decreased remarkably compared with control (Fig. 3b). To assess the cell population by its size, we performed flow cytometry analysis, and observed that smaller cell population was increased by knockdown of AQP4 compared with control (Fig. 3c). Forward scatter (FSC) data showed significant decrease in the size of AQP4 shRNA-transfected myotubes. Interestingly, side scatter (SSC) data revealed no difference in the complexity or granularity of the cell, suggesting that the reduced size of cells is not caused by the change in the internal complexity of cells (Fig. 3d,e).

### RCT-induced reduction of AQP4 protein levels correlates with upregulation of atrogin 1

**Figure 4:**
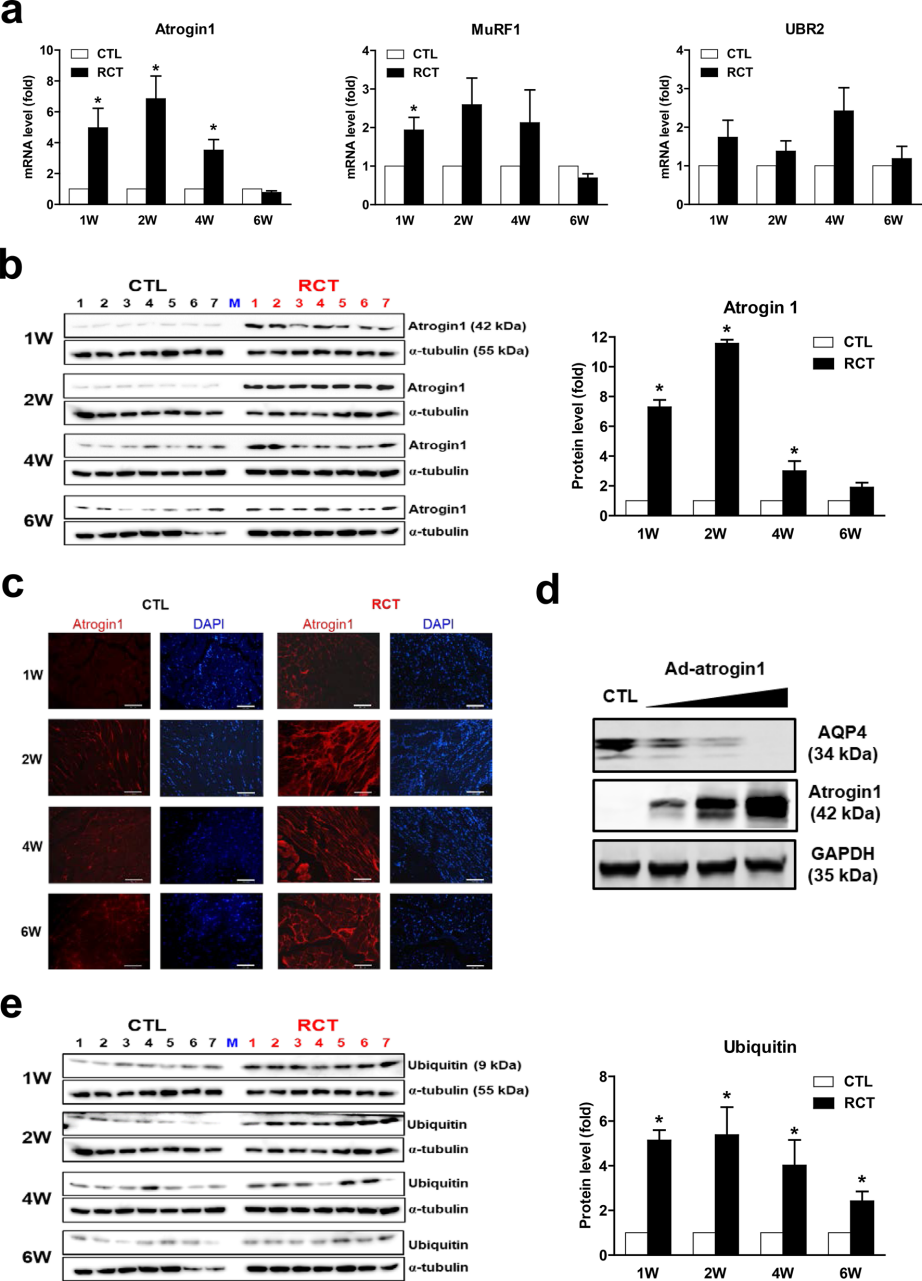
Induction of atrogin 1 mRNA and protein expression by rotator cuff tear (RCT). (**a**) Expression of transcripts encoding the E3 ubiquitin-protein ligases atrogin 1, MuRF1, and UBR2 in injured muscle after RCT (n = 12 per group). (**b**) Atrogin 1 protein expression in muscle isolated at different stages after RCT (n = 7 per group). Densitometric analyses of the western blots are shown on the right. Data represent means ± standard errors of the means. *P < 0.05. (**c**) Immunofluorescence (IF) microscopy analyses of muscle isolated at different stages after RCT. The red signal represents atrogin 1 protein. The negative control (secondary antibody only) for the IF showed no specific signal (data not shown). (**d**) Loss of AQP4 protein by overexpression of atrogin 1. C2C12 cells were treated with an adenovirus expressing atrogin 1 [Ad-atrogin 1; multiplicity of infection (MOI) = 50, 100, 200] for 48 h, and western blot analysis was performed with each designated antibody. (**e**) Monomeric ubiquitin protein expression in muscle cells isolated at different stages after RCT.

Muscle atrophy is caused by destruction of the component proteins of skeletal muscle, and protein degradation is typically mediated by E3 ubiquitin ligases^14^. To examine whether loss of AQP4 is directly related to activation of E3 ligases, we measured the expression levels of *atrogin 1*, *MuRF1*, and *Ubr2* genes, which encode representative regulators of ubiquitin-mediated protein degradation in skeletal muscle. As shown in Fig. 4a, among the E3 ligase genes tested, expression of *atrogin 1* was most strongly induced by RCT, although it had returned to a normal level after 6 weeks post-surgery. Western blot analysis also revealed that RCT led to remarkably upregulated atrogin 1 expression, corroborating our RT-qPCR data (Fig. 4b). Immunofluorescence staining with an anti-atrogin 1 antibody showed this protein to be localized in the nucleus in control muscle, but predominantly in the cytoplasm in myocytes of muscle injured by RCT (Fig. 4c). In addition, atrogin 1 overexpression caused significant reduction in AQP4 protein levels suggesting the direct correlation between upregulation of atrogin 1 and loss of AQP4 protein (Fig. 4d).

The major role of E3 ligases is to recognize and transfer ubiquitin to a specific substrate protein, which is consequently enzymatically degraded, for instance, by the 26S proteasome. To ascertain whether upregulation of atrogin 1 by RCT is directly linked to increased ubiquitin expression, we measured protein levels by western blot analysis. As shown in Fig. 4e, levels of monomeric ubiquitin protein were significantly increased by RCT, with the highest increase being observed 2 weeks after surgery. Immunofluorescence staining also revealed strong ubiquitin protein expression (Supplementary Fig. 3). Taken together, these results indicated that RCT upregulates atrogin 1 expression and augments ubiquitin levels, which may lead to loss of AQP4 protein in injured muscle and cause muscle atrophy.

### Atrogin 1 expression is regulated by HMGB1 in injured muscle after RCT

**Figure 5:**
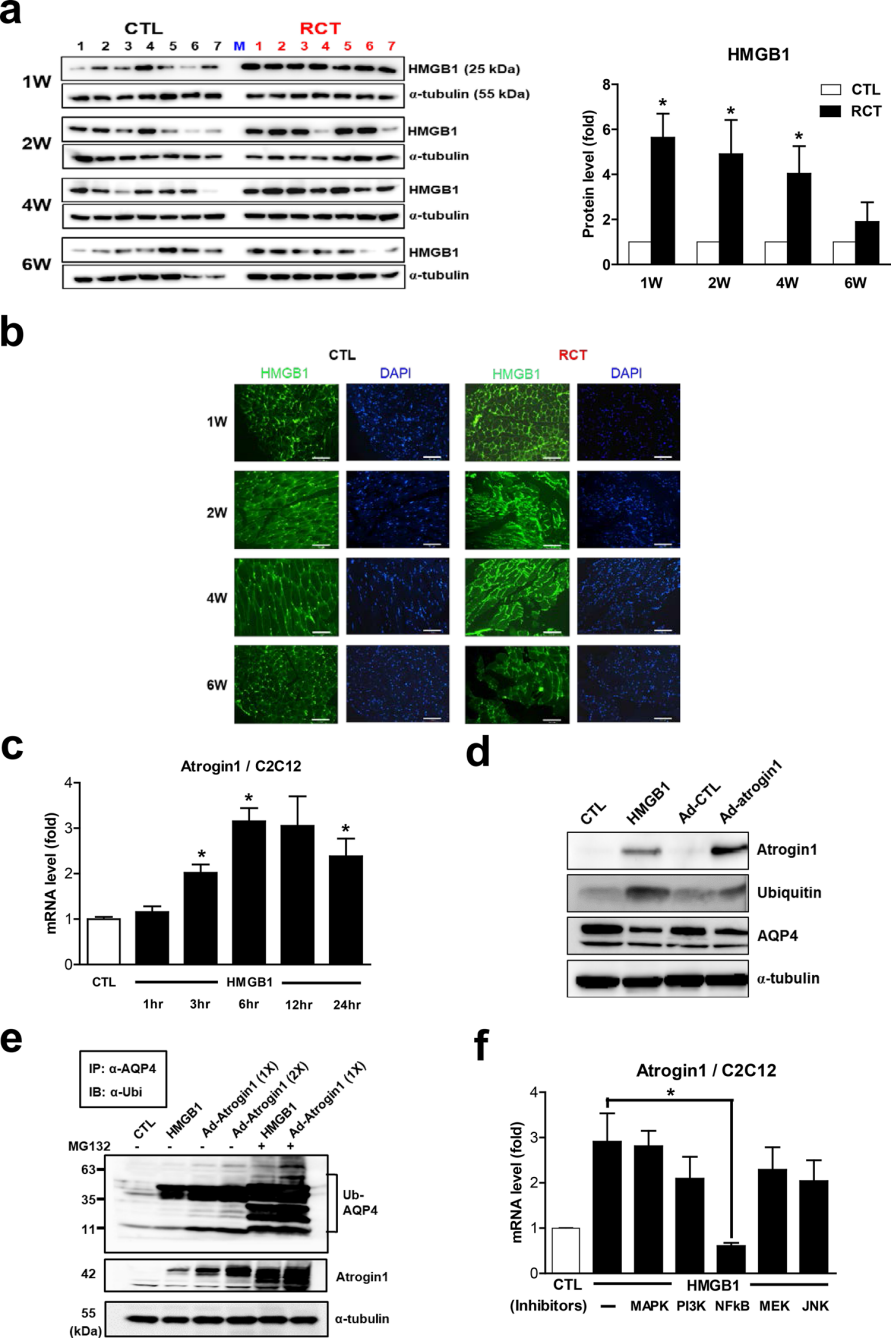
Increased levels of HMGB1 protein in muscle injured by rotator cuff tear (RCT). (**a**) Expression level of HMGB1 protein in muscle isolated at different stages after RCT (n = 7 per group). Densitometric analyses of the western blots are shown on the right. Data represent means ± standard errors of the means. *P < 0.05. (**b**) Immunofluorescence (IF) microscopy analyses of muscle cells isolated at different stages after RCT. The green signal represents HMGB1 protein. The negative control (secondary antibody only) for the IF showed no specific signal (data not shown). (**c**) Expression of atrogin 1 mRNA in C2C12 myotubes treated with 200 ng/mL recombinant mouse HMGB1 for the indicated time periods. (**d**) Atrogin 1, ubiquitin, and AQP4 protein levels in C2C12 myotubes treated with 200 ng/mL HMGB1 and an adenovirus expressing atrogin 1 [Ad-atrogin 1; multiplicity of infection (MOI) = 100] for 48 h. (**e**) Ubiquitination of AQP4 caused by HMGB1 and atrogin 1. C2C12 myotubes were treated with 200 ng/mL HMGB1 and Ad-atrogin 1 (MOI = 100) with or without MG132. After 48 h of incubation, cell lysates were prepared for a ubiquitination assay using anti-AQP4 and anti-ubiquitin antibodies. (**f**) Expression of atrogin 1 mRNA in C2C12 myotubes exposed to 5 μM SB203580 (a p38 MAPK inhibitor), LY294002 (a PI3K inhibitor), BAY 11-7082 (an NF-κB inhibitor), PD98059 (an MEK inhibitor), or SP600125 (a JNK inhibitor). Cells were exposed to each inhibitor for 1 h prior to treatment with 200 ng/mL HMGB1 for 24 h.

In the process of finding the potent upstream regulators of RCT-induced atrogin 1 expression, we speculated that damage-associated molecular patterns (DAMPs) could be the possible candidates. Among such molecules, HMGB1 attracted our attention because it is released from injured or dying cells in damaged muscles and is involved in activating the innate immune response^28^. As shown in Fig. 5a, HMGB1 protein expression was significantly upregulated in injured muscle. In particular, its levels were dramatically increased during the acute phase of RCT, suggesting that it is directly released from damaged myocytes following RCT. In accordance with this finding, immunofluorescence staining revealed that HMGB1 was predominantly present in nuclei in non-injured muscle, with a greater degree of cytoplasmic localization being observed following RCT (Fig. 5b). These results indicated that RCT augments HMGB1 protein levels and that this DAMP might be a potent regulator of atrogin 1 expression.

### The RCT–HMGB1–atrogin 1 axis induces loss of AQP4 in injured muscle via ubiquitination

To examine whether HMGB1 directly regulates atrogin 1 gene expression, we treated C2C12 myotubes with recombinant HMGB1 protein. As shown in Fig. 5c, atrogin 1 mRNA levels were significantly increased after HMGB1 administration. Western blot analysis revealed that protein levels of both atrogin 1 and ubiquitin were prominently augmented and those of AQP4 were reduced by HMGB1 treatment, as well as by atrogin 1 overexpression (Fig. 5d). To assess whether the loss of AQP4 protein was caused by its ubiquitination, we performed a ubiquitination assay, which revealed that both HMGB1 administration and atrogin 1 overexpression resulted in AQP4 ubiquitination and proteasome degradation (Fig. 5e). These results demonstrated that HMGB1-mediated atrogin 1 expression leads to loss of AQP4 by ubiquitination.

HMGB1 acts through multiple surface receptors, including TLR2, TLR4, and RAGE. Importantly, interaction between HMGB1 and TLR4 results in upregulation of NF-κB, which leads to increased production and release of cytokines as part of various immune responses^22^. Therefore, we subjected *Tlr4*-KO mice to RCT to investigate whether loss of AQP4 is mediated via HMGB1-TLR4 signaling. However, contrary to our expectation, AQP4 protein levels were significantly reduced in injured muscle in *Tlr4*-KO as well as wild type mice after RCT (Supplementary Fig. 4). This result indicated that TLR4 is not the receptor through which HMGB1 induces elevated atrogin 1 expression leading to AQP4 ubiquitination in injured muscle. To identify the signaling molecule(s) directly involved in HMGB1-induced atrogin 1 expression within myocytes, we examined the effects of various signaling inhibitors. As shown in Fig. 5f, HMGB1-induced atrogin 1 mRNA expression in C2C12 cells was significantly suppressed by the NF-κB inhibitor, BAY 11-7082, suggesting that NF-κB is the major mediator of HMGB1 signaling via an unidentified receptor in myocytes. Taken together, these results suggested that, through NF-κB activation, HMGB1 directly regulates atrogin 1 gene expression, which causes AQP4 ubiquitination in myocytes after RCT.

## Discussion

Considering that it is highly expressed in skeletal muscle, AQP4 may play an important role in maintaining myocyte water homeostasis and cell volume. In the present study, we found that both human and mouse AQP4 protein levels were significantly decreased in muscle injured by RCT. The loss of AQP4 was directly caused by atrogin 1-mediated ubiquitination, and the RCT-induced atrogin 1 expression was found to be regulated by HMGB1 via NF-κB signaling. Although the size and the number of myotubes were remarkably reduced by knockdown of AQP4, we were unable to demonstrate a direct causal relationship between loss of AQP4 protein and myocyte atrophy in injured muscle after RCT. However, it is well known that AQP4 is involved in the proliferation, survival, migration, and neuronal differentiation of adult neural stem cells^29^. Moreover, its absence results in aberrant expression of proteins that are involved in energy metabolism and calcium homeostasis in skeletal muscle^30^. A recent paper has also shown that levels of AQP4 protein are markedly decreased in muscles atrophied by denervation due to defective functional and structural relationships with α1-syntrophin and transient receptor potential vanilloid 4^31^. In addition, it has been reported that AQP4 loss in skeletal muscle correlates with muscular dystrophy^32^, and the expression levels of AQP4 and α1-syntrophin are maintained in hypertrophied muscles^33^. In the current study, loss of AQP4 protein clearly correlated with myocyte shrinkage in injured muscle following RCT. Thus, our results and those of others support the conclusion that AQP4 loss is associated with substantial muscle atrophy.

The maintenance of muscle mass is regulated by a balance between protein synthesis and degradation, with muscle atrophy occurring when the latter exceeds the former^13,14^. To date, three mechanisms underlying muscle atrophy have been identified: the UPS; apoptosis via caspase signaling; and autophagy^34^. In our study, we found that loss of AQP4 was due to the proteasomal degradation of this protein mediated by atrogin 1-dependent UPS activity. Atrogin 1, also known as F-box protein 32 (FBXO32), was initially identified as a muscle-specific E3 ubiquitin ligase required for skeletal muscle atrophy^35^. AQP4 is a selective water channel located on the plasma membrane of skeletal muscle myofibers, and transiently interacts with regulatory proteins^36^. Moreover, it has been reported that AQP4 can be targeted for degradation by the ubiquitin-dependent proteasome pathway^37^. Using a ubiquitination assay, we demonstrated here that overexpression of atrogin 1 induces AQP4 ubiquitination, which is a novel finding indicating that direct targeting of AQP4 by atrogin 1 underlies RCT-induced muscle atrophy.

The molecular mechanisms affecting atrogin 1 expression in injured muscle have been extensively studied, yet little is known of whether and how inflammation modulates these processes. Upregulation of several genes associated with inflammation in muscle has been demonstrated in mouse model of muscle injury^38^, including in our own previous study^27^. In the present work, we examined the effects of inflammatory cytokines on atrogin 1 expression; however, neither LPS nor IL-6 exhibited a substantial effect on this protein (data not shown). We therefore turned our attention to the possibility that HMGB1 could be a potent upstream regulator of atrogin 1 expression in RCT. HMGB1 is an endogenous molecule that is normally localized to the nucleus, where it participates in the organization of DNA and nucleosomes and regulates gene transcription. However, upon cell injury that causes plasma membrane rupture or death, HMGB1 freely diffuses from the nucleus, translocating to the cytoplasm^22^. As our present results show, this molecule was rapidly upregulated in injured muscle resulting from RCT, and directly increased atrogin 1 expression at both the mRNA and protein levels. Notably, our immunofluorescence microscopy assay revealed that HMGB1 was predominantly present in the nucleus in the control group but was abundant in the cytoplasm of cells in RCT-injured muscle. This novel finding suggested that HMGB1 is released from injured or dying cells in damaged muscles and plays a crucial role in activating atrogin 1 expression.

Despite our findings concerning the mechanisms by which muscle injury affects HMGB1-dependent atrogin 1 expression, the precise signaling pathways involved remain to be elucidated in future studies. To date, several HMGB1 receptors have been identified^22^. TLR4 is representative of receptors associated with HMGB1 signaling, and acts as a sentinel of inflammatory response induction in skeletal muscle^20^. In the present study, based on our experiment using *Tlr4*-KO mice, the effects of HMGB1 on injured myocytes after RCT did not appear to be mediated by TLR4, suggesting that the interaction between this receptor and TLR4 is not involved in atrogin 1 expression. However, HMGB1-induced atrogin 1 mRNA expression was significantly suppressed by an NF-κB inhibitor. A recent study demonstrated that activin A upregulates atrogin 1 expression and triggers C2C12 myotube atrophy via activation of p38β MAPK^39^. In contrast, in our study, HMGB1-dependent atrogin 1 expression was not altered by administration of a MAPK inhibitor, suggesting that the observed effects of RCT-induced HMGB1 expression do not rely on MAPK signaling. Therefore, considering the fact that HMGB1 signaling via other receptors, such as TLR2 and RAGE, also leads to MAPK or NF-κB activation, HMGB1 may exhibit receptor-specificity during its role in skeletal muscle.

In conclusion, we demonstrated here, for the first time, that atrogin 1-dependent degradation of AQP4 is associated with muscle atrophy, and that HMGB1 is a potent regulator of atrogin 1 expression in injured muscle after RCT. Our novel findings will help other researchers to better understand the pathways contributing to injury-induced muscle atrophy or loss of muscle mass. In this context, identifying the specific receptor(s) of HMGB1, a potent regulator of atrogin 1 expression, could be useful in clinical applications.

## Methods

### Human tissue preparation

All patients underwent preoperative magnetic resonance imaging (MRI, 3.0-T, Signa HDx; GE Healthcare, Pewaukee, Wisconsin, USA), and the fatty infiltration of each rotator cuff muscle (supraspinatus, infraspinatus, and subscapularis) was graded according to the criteria established by Goutallier et al.^23^, by an experienced musculoskeletal radiologist who was blinded to this study. Human tissue samples (supraspinatus muscle and deltoid muscle as controls, n = 9, each) were acquired arthroscopically (supraspinatus muscle (3 × 3 mm): 1 cm from the musculotendinous junctions; deltoid muscle (3 × 3 mm): from the anterior portion of the deltoid at a footprint level) using an arthroscopic punch through the lateral portal during an arthroscopic rotator cuff repair. The intact deltoid muscle was used for the control muscle instead of intact supraspinatus muscle due to ethical issues. Samples were used for tissue staining and western blot analysis, respectively.

### Animals and mouse RCT model

Male 8-week-old C57BL/6 mice and *Tlr4*-knockout (KO) mice were purchased from Orient Bio Inc. (Seongnam, Korea) and the Jackson Laboratory (Bar Harbor, ME, USA), respectively, and kept in a specific-pathogen-free facility. Prior to experiments, the mice were acclimatized to a 12-h/12-h light/dark cycle at 22 ± 2 °C for 1 week and allowed unlimited access to food and water. We generated the mouse RCT model as described previously^27^. Briefly, C57BL/6 mice were randomly divided into four groups (1, 2, 4, and 6 weeks after RCT; n = 15/group) and subjected to unilateral complete supraspinatus tendon transection under anesthesia. The mice were sacrificed at 1, 2, 4, and 6 weeks after surgery, and the supraspinatus muscles were completely separated from the scapular fossa. The muscle samples were used for total RNA or protein extraction (n = 12 per group) and immunohistological analyses (n = 3 per group). The contralateral supraspinatus muscle was used as a control.

### Reverse-transcription quantitative PCR (RT-qPCR) analysis

RT-qPCR analysis was performed as described previously^27^. The primer sequences are given in Table 1.

**Table 1:** The sequences of the primers.

Gene full name	Gene symbol (mouse)	Sequences; forward (F)/reverse (R)
Aquaporin 4	Aqp4	(F) 5′-TTGCTTTGGACTCAGCATTG-3′ (R) 5′-AACCAGGAGACCATGACCAG-5′
F-box protein 32 (Atrogin 1)	Fbxo32	(F) 5′-ATGCACACTGGTGCAGAGAG-3′ (R) 5′-TGTAAGCACACAGGCAGGTC-4′
Tripartite motif-containing 63 (MuRF1)	Trim63	(F) 5′-ACCTGCTGGTGGAAAACATC-3′ (R) 5′-AGGAGCAAGTAGGCACCTCA-5′
Ubiquitin protein ligase E3 component n-recognin 2	Ubr2	(F) 5′-ATAATACCGATCCCCGAAGG-3′ (R) 5′-CTGGATGGTGTATGCACAGG-6′
Interleukin 1 beta	Il-1b	(F) 5′-GAATCTATACCTGTCCTGTG-3′ (R) 5′-ACGGATTCCATGGTGAAGTC-3′
Interleukin 6	Il-6	(F) 5′-AACGATGATGCACTTGCAGA-3′ (R) 5′-GAGCATTGGAAATTGGGGTA-3′
CCAAT/enhancer binding protein (C/EBP), alpha	Cebpa	(F) 5′-AAGAAGTCGGTGGACAAGAAC-3′ (R) 5′-GTCATTGTCACTGGTCAGCTC-3′
Peroxisome proliferator activated receptor gamma	Pparg	(F) 5′-CGGTTTCAGAAATGCCTTGC-3′ (R) 5′-ATCTCCGCCAACAGCTTCTC-3′
Myogenic differentiation 1	Myod1	(F) 5′-CAAGCGCAAGACCACCAACG-3′ (R) 5′-ATATAGCGGATGGCGTTGC-3′
Myogenic factor 5	Myf5	(F) 5′-CCTCATGTGGGCCTGCAAAG-3′ (R) 5′-CATTCCTGAGGATCTCCACC-3′
Actin, beta	Actb	(F) 5′-TCTGGCACCACACCTTCTAC-3′ (R) 5′-TCGTAGATGGGCACAGTGTGG-3′

### Western blot analysis

Whole cell extracts from isolated muscle tissue or cultured cells were prepared using RIPA buffer (Elpis-Biotech, Korea). Proteins from the whole cell lysates were separated by 15% SDS-PAGE and transferred to nitrocellulose membranes. The membranes were cut into 3 parts by the size; 6–30 kDa for ubiquitin (9 kDa) and HMGB1 (25 kDa), 30–53 kDa for AQP4 (34 kDa) and atrogin1 (42 kDa), 53–170 kDa for α-tubulin (55 kDa) and MYH (120 kDa) to ensure the same experimental conditions instead of membrane duplication. Thus, all the images of α-tubulin in each panel was used as the same images of loading control. Membranes were probed with each antibody; anti-AQP4 (NBP1-87679, Novus Biologicals, Littleton, CO, USA), anti-atrogin 1/MAFbx (F-9) (sc-166806, Santa Cruz Biotechnology, Dallas, TX, USA), anti-ubiquitin (P4D1) (sc-8017, Santa Cruz Biotechnology), anti-HMGB1 (ab79823, Abcam, Cambridge, MA, USA), anti-myosin heavy chain (MYH) (sc-376157, Santa Cruz Biotechnology), and anti-α-tubulin (ab7291, Abcam) antibody. Immunoreactive proteins were visualized using an Amersham ECL kit (GE Healthcare, NJ, USA) and assessed by using the LAS-3000 Image analyzer (Fuji Film, Tokyo, Japan). The protein amounts were assessed by densitometry using Image J software (National Institutes of Health, Bethesda, MD, USA).

### Histological analysis and immunofluorescence microscopy

Histological and immunofluorescence microscopy analyses were performed as described previously^27^. The resulting immune complexes were visualized with a goat anti-rabbit IgG H&L (Alexa Fluor 488) (ab150077, Abcam) or goat anti-mouse IgG H&L (Alexa Fluor 594) (ab150116, Abcam) secondary antibody. Nuclei were stained with 4′,6-diamidino-2-phenylindole (VECTASHIELD Hardset Antifade Mounting Medium, H-1500, Vector Laboratories, Inc., Burlingame, CA, USA). Images were acquired using an upright fluorescence microscope (BX61-32FDIC, Olympus, Tokyo, Japan).

### Cell culture, reagents, and adenovirus

C2C12 mouse myoblasts were cultured in Dulbecco’s modified Eagle’s medium (DMEM) (Corning, NY, USA) supplemented with 10% fetal bovine serum and antibiotics at 37 °C in a humidified atmosphere containing 5% CO_2_. To differentiate into myotubes, C2C12 cells were maintained in DMEM supplemented with 2% horse serum for 72 h, and the medium was replaced every other day. Recombinant mouse HMGB1 (BioLegend Inc., San Diego, CA, USA) was dissolved in phosphate-buffered saline (PBS). Specific inhibitors of p38 MAPK (SB203580), PI3K (LY294002), MEK (PD98059), JNK (SP600125), and NF-κB (BAY 11-7082) were purchased from Cell Signaling Technology (Danvers, MA, USA) and dissolved in the recommended solvents. An adenovirus expressing atrogin 1 (Ad-atrogin 1/FBXO32) was kindly provided by Prof. J.-S. Chun of the School of Life Sciences, Gwangju Institute of Science and Technology, Gwangju, Republic of Korea^40^.

### Producing lentiviral particles and transfection of AQP4 shRNA

For stable loss-of-function experiments, lentiviral particles for AQP4 shRNA were generated and transfected by a protocol described in a website (https://www.addgene.org/tools/protocols/plko). shRNA specific to the mouse AQP4 gene, as well as the control construct, pLKO-GFP, were commercially purchased (AQP4 MISSION shRNA, SHCLNG-NM_009700, Sigma-Aldrich, St. Louis, MO, USA). The sequence of the shRNA targeting the AQP4 mRNA was 5′-CCGGCAATTGGACATTTGTTTGCAACTCGAGTTGCAAACAAATGTCCAATTGTTTTTG-3′.

### Cell size and complexity analysis by flow cytometry

C2C12 cells were seeded at a density of 1 × 10^5^ cells per well of 6-well plates and incubated for 24 h, and then, the cells were differentiated into myotubes in DMEM supplemented with 2% horse serum for an additional 72 h. At 24 h after differentiation, the cells were transfected with shAQP4. The cells were harvested, washed with PBS twice, and then, suspended with 0.5 mL of PBS. The size and granularity (complexity) of cells were determined by forward and side scatter (FSC and SSC) gating, respectively, using flow cytometry on a FACScalibur instrument and analyzed with CellQuest software (BD Bioscience, San Jose, CA, USA).

### Immunoprecipitation and ubiquitination assay

C2C12 myotubes were treated with HMGB1 and Ad-atrogin 1 for 48 h in the presence or absence of MG132, and the cell lysates were used for ubiquitination assay. Briefly, the cell lysates were incubated with an anti-AQP4 antibody for 12 h at 4 °C under gentle rotation. Protein A-coupled Sepharose beads (Abcam) were washed then times with PBS and mixed with the lysate-antibody mixture for 4 h at 4 °C. The antigen–antibody complex was eluted from the beads by heating samples in loading buffer and used for immunoblotting with an anti-ubiquitin (Ub) antibody.

### Statistical analysis

Data are expressed as means ± standard errors. Differences between the sham and tear side were assessed using *t*-tests (α = 0.05) in GraphPad Prism 5.01 (GraphPad Software, La Jolla, CA, USA). Differences were considered significant at *P* < 0.05.

### Ethical approval

This study was approved by the institutional review board (IRB) of Konkuk University Hospital (IRB no. KUH1060151), and written informed consent was obtained from all patients. In addition, all animal experiments were approved by the Institutional Animal Care and Use Committee of Konkuk University (IACUC No. KU17122) and were performed in accordance with the ethical standards laid down in the 1964 Declaration of Helsinki and its later amendments.

## Supplementary information

Supplementary Information.
