# Supplementary material for: Atrogin1-induced loss of aquaporin 4 in myocytes leads to skeletal muscle atrophy
Source: Sci Rep. 2020 Aug 25;10:14189. doi: 10.1038/s41598-020-71167-8 (PMC7447774; doi:10.1038/s41598-020-71167-8)

Atrogin1-induced loss of aquaporin 4 in myocytes leads to skeletal muscle atrophy

Seok Won Chung[1], Ja-Yeon Kim[1], Jong Pil Yoon[2], Dong Won Suh[3], Woo Jin Yeo[3] and Yong-Soo Lee[1, 3]*

[1] Department of Orthopedic Surgery, Research Institute of Medical Science, Konkuk University School of Medicine, Seoul, Republic of Korea

[2] Department of Orthopedic Surgery, School of Medicine, Kyungpook National University, Daegu, Republic of Korea

[3] Joint Center, Barunsesang Hospital, #75-5, Yatap-ro, Seongnam-si, 13497, Gyeonggi-do, Republic of Korea

* Correspondence:

Yong-Soo Lee, PhD

Joint Center, Barunsesang Hospital, #75-5, Yatap-ro, Seongnam-si, 13497, Gyeonggi-do, Republic of Korea

Tel.: +82-31-750-9937

Fax: +82-31-783-9631

E-mail: hrcyslee@yahoo.co.kr

Supplementary Fig. 1

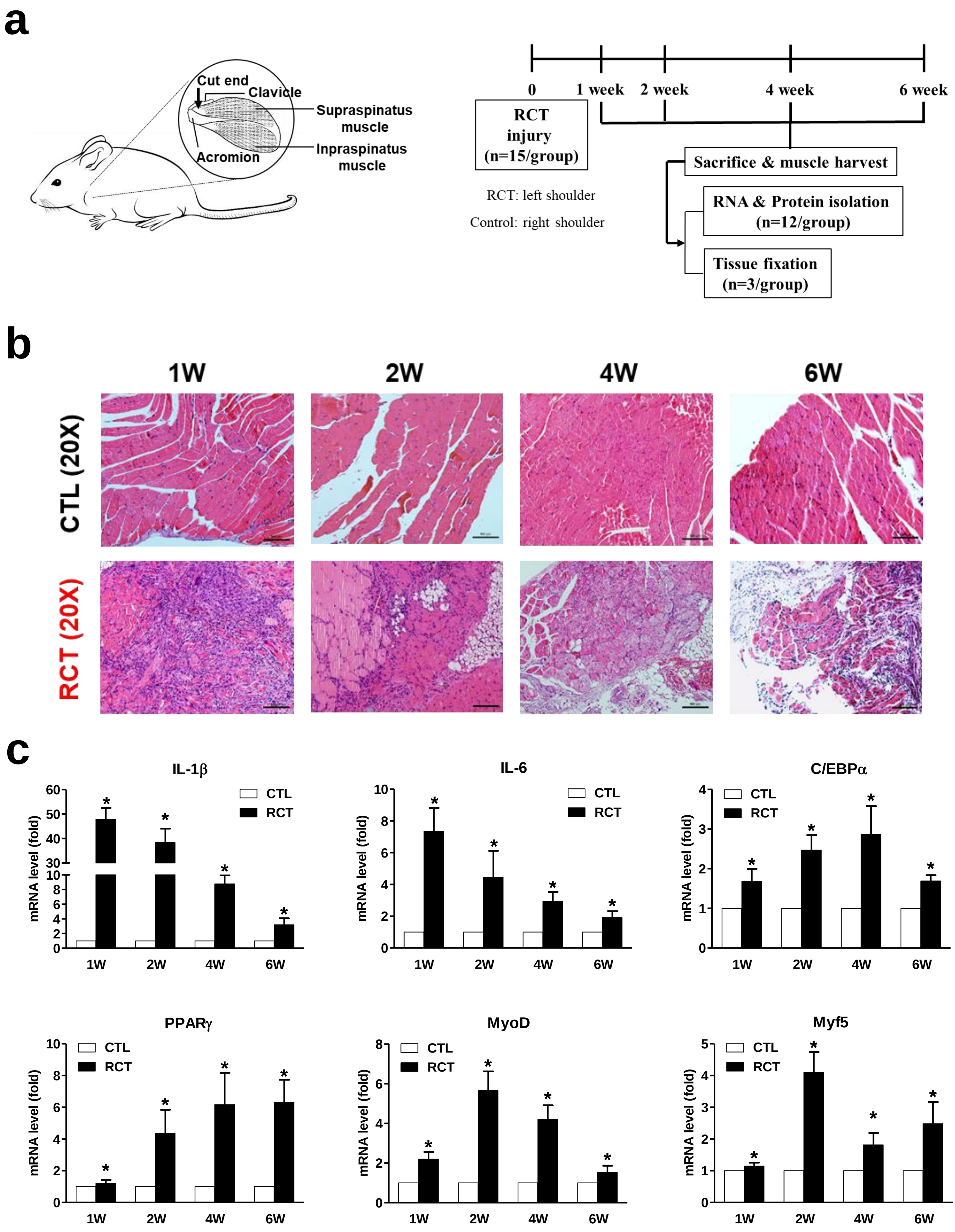

Supplementary Fig. 2

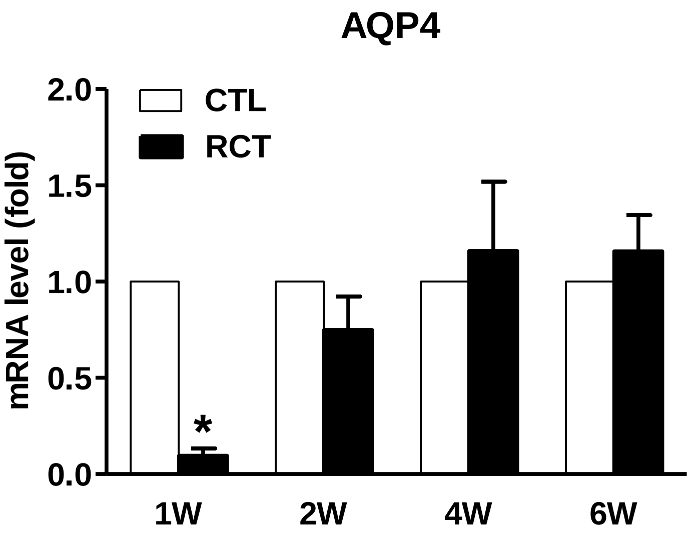

Supplementary Fig. 3

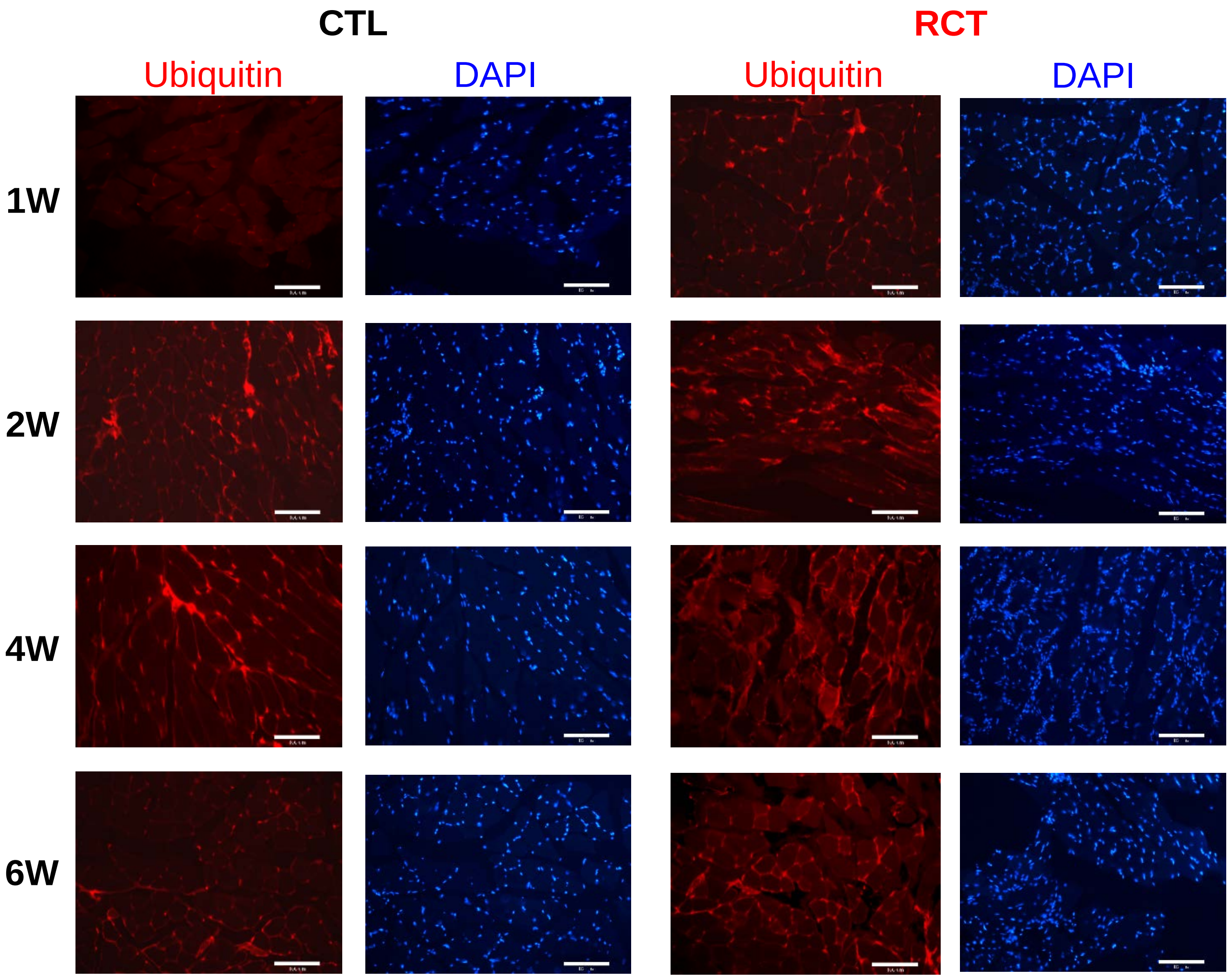

Supplementary Fig. 4

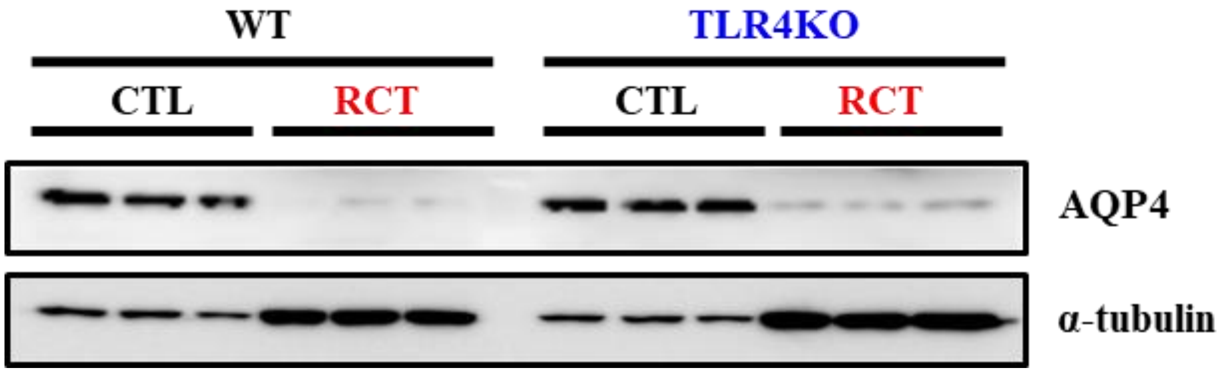

Supplementary Fig. 1 Morphological and genetical changes in muscles resulting from rotator cuff tear (RCT)-induced inflammation, fatty infiltration, and atrophy. (a) A mouse RCT model was generated as described, and mice were sacrificed 1, 2, 4, and 6 weeks after RCT (n = 15 per group). (b) Histological analyses of the isolated muscle after RCT surgery and hematoxylin and eosin (H&E) staining (n = 3 per group). Magnification, 200×. Scale bar, 100 μm. The cracked appearance of the muscle appears to have been caused by infiltration of injured muscle by fat. (c) Gene expression changes after rotator cuff tear (RCT) in mice (n = 12 per group). The supraspinatus muscles were completely removed from the scapular fossa, and then, used for total RNA isolation. Reverse-transcription quantitative PCR was performed with primers targeting mouse *Il-1b* and *Il-6, Cebpa*, *Pparg*, *Myod1* and *Myf5*. The contralateral supraspinatus muscle was used as a control. *$P < 0.05$

Supplementary Fig. 2 Expression level of *Aqp4* mRNA in muscle cells isolated at different stages after RCT (n = 12 per group). The supraspinatus muscles were completely removed from the scapular fossa, and then, used for total RNA isolation. Reverse-transcription quantitative PCR was performed with primers targeting mouse *Aqp4*. The contralateral supraspinatus muscle was used as a control. *$P < 0.05$

Supplementary Fig. 3 Upregulated ubiquitin expression following rotator cuff tear (RCT). Immunofluorescence microscopy analyses of muscle isolated at different stages after RCT (n = 3 per group). The red signal represents ubiquitin protein. Magnification, 200×. Scale bar, 100 μm.

Supplementary Fig. 4 AQP4 protein expression in muscles isolated from wild type (WT) and Tlr4-knockout (KO) mice at 2 weeks after RCT. A mouse RCT model was generated in WT and Tlr4-KO mice, and mice were sacrificed 2 weeks after RCT (n = 3 per group). Western blot analysis was performed using anti-AQP4 antibody.

Supplementary Table1

The information of enrolled patients

Patient #	Sex	Age	Goutallier grade (Supraspinatus)	Visual occupation ratio
1	M	41	2	63.10%
2	M	45	2	72.90%
3	M	45	2	67.30%
4	M	51	3	53.20%
5	M	49	2	63.40%
6	M	51	1	76.30%
7	M	52	2	67.80%
8	M	53	2	62.70%
9	M	54	3	41.60%

Figure 1

Fig. 1 f AQP4

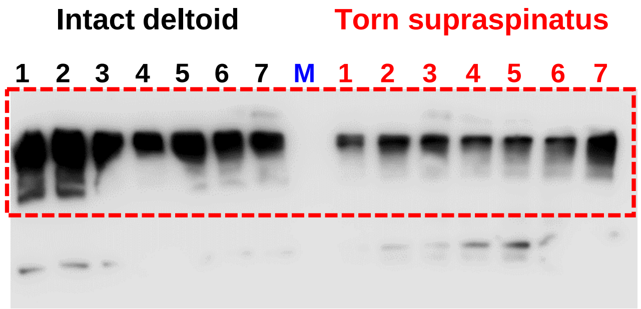

Fig. 1 f α-tubulin

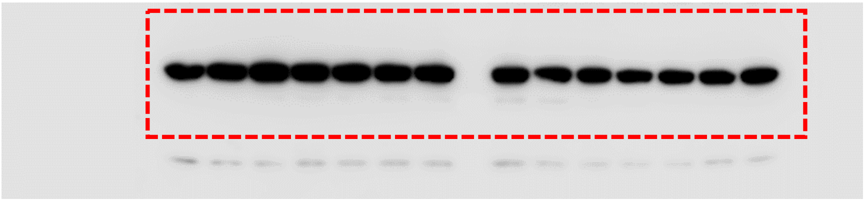

Figure 2

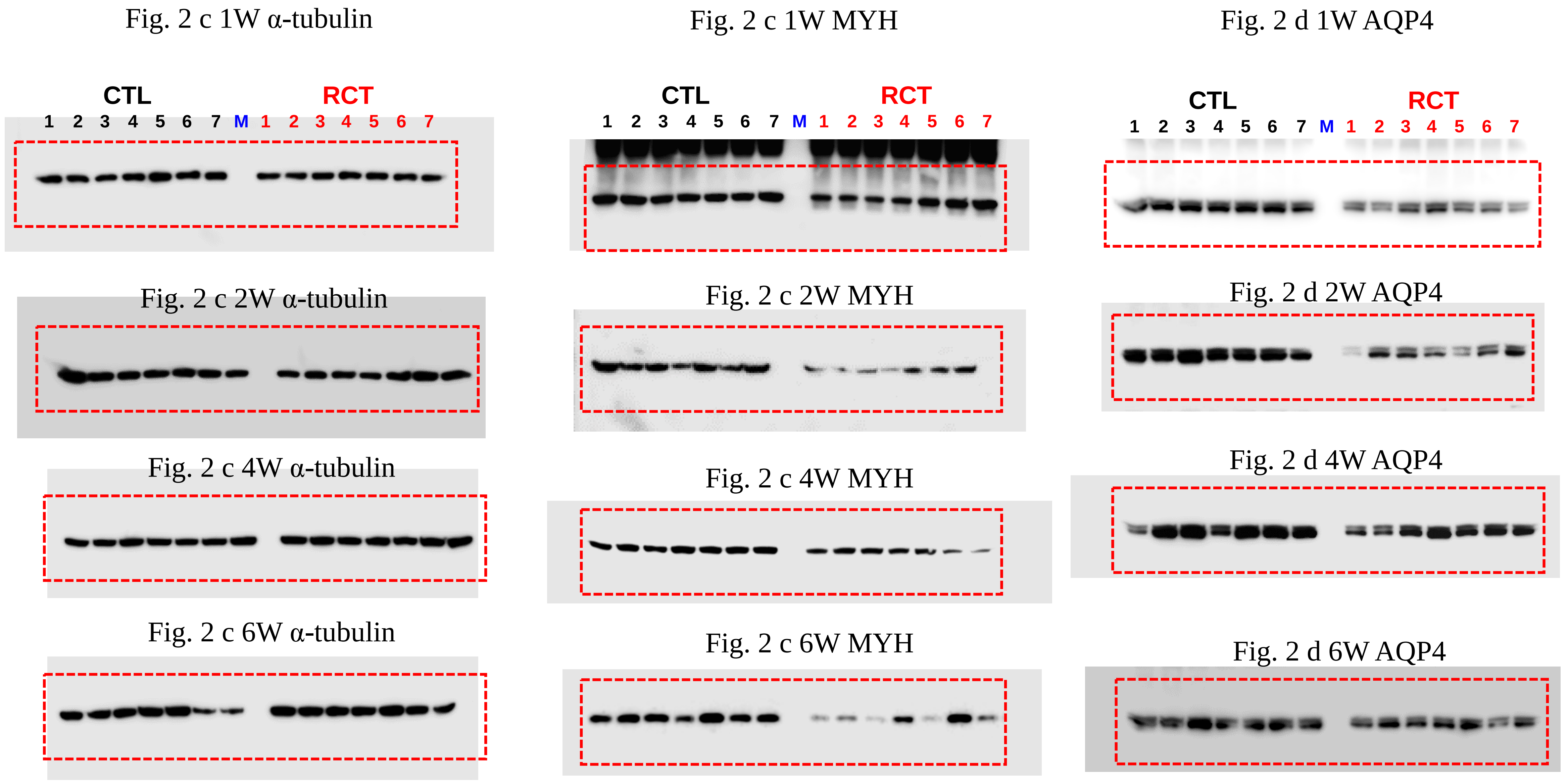
Fig. 2 c 1W α-tubulin
Fig. 2 c 1W MYH
Fig. 2 d 1W AQP4
CTL
RCT
1 2 3 4 5 6 7 M 1 2 3 4 5 6 7
Fig. 2 c 2W α-tubulin
Fig. 2 c 2W MYH
Fig. 2 d 2W AQP4
Fig. 2 c 4W α-tubulin
Fig. 2 c 4W MYH
Fig. 2 d 4W AQP4
Fig. 2 c 6W α-tubulin
Fig. 2 c 6W MYH
Fig. 2 d 6W AQP4

Figure 3

Fig. 3 a AQP4

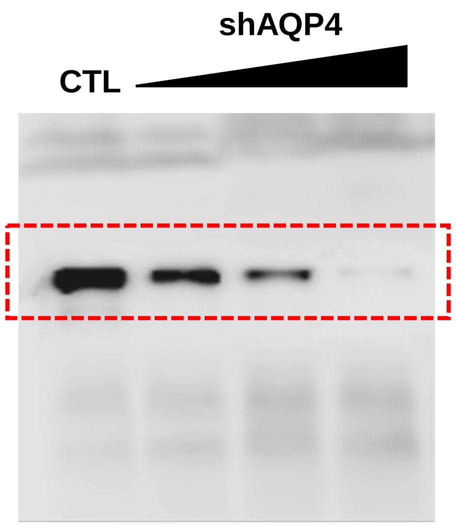

Fig. 3 a α-tubulin

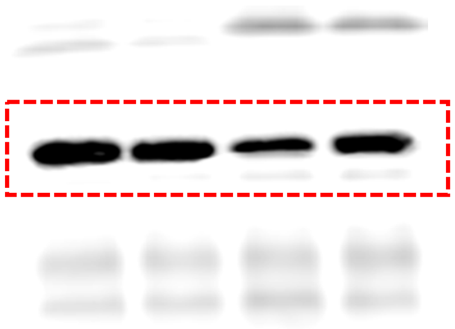

Figure 4

Fig. 4 b 1W Atrogin1

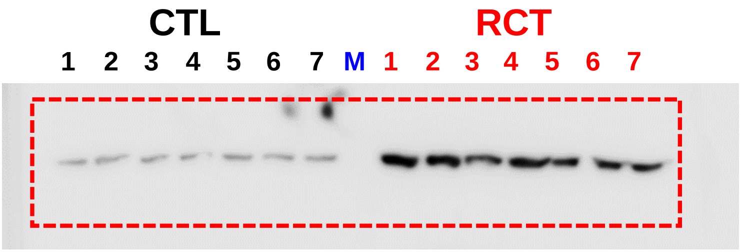

Fig. 4 b 2W Atrogin1

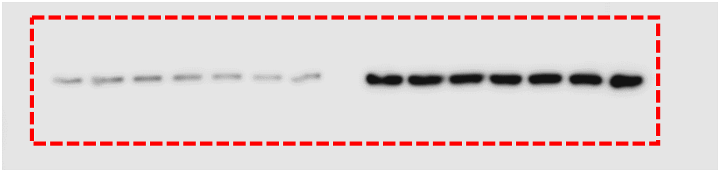

Fig. 4 b 4W Atrogin1

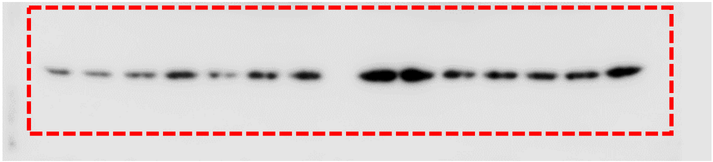

Fig. 4 b 6W Atrogin1

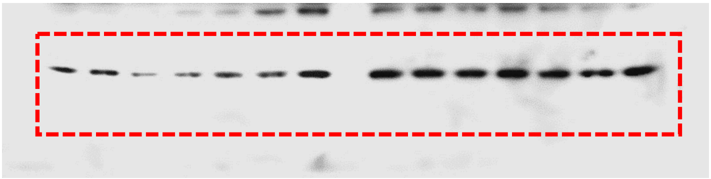

Fig. 4 d AQP4

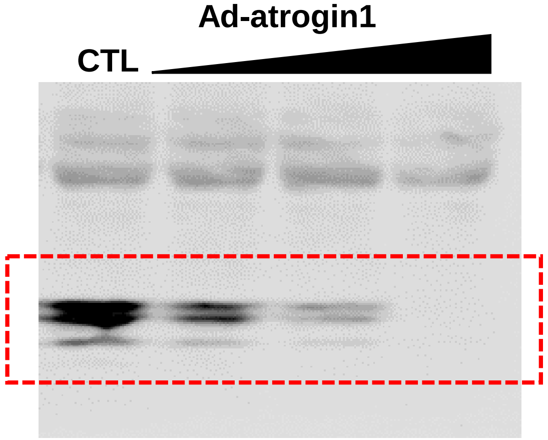

Fig. 4 d Atrogin1

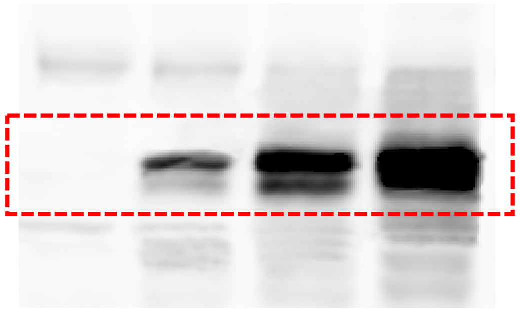

Fig. 4 d GAPDH

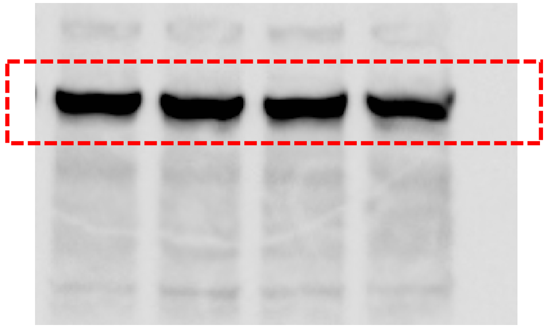

Fig. 4 e 1W Ubiquitin

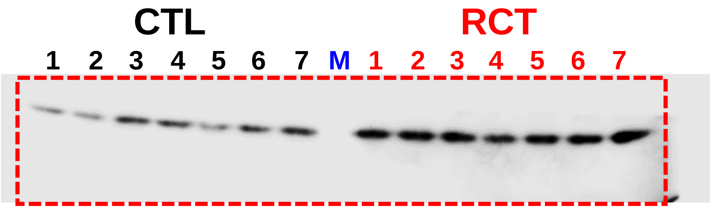

Fig. 4 e 2W Ubiquitin

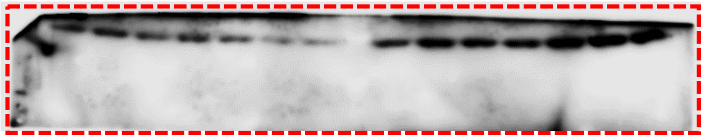

Fig. 4 e 4W Ubiquitin

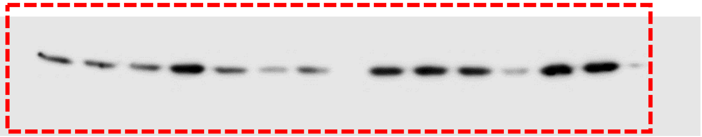

Fig. 4 e 6W Ubiquitin

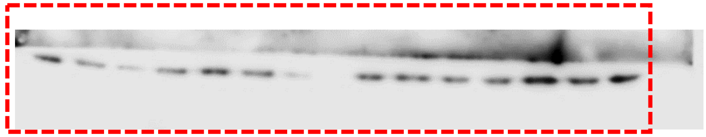

Figure 5

Fig. 5 a 1W HMGB1

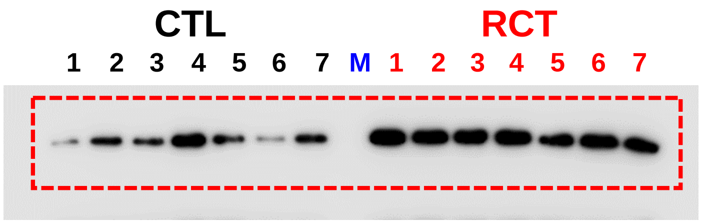

Fig. 5 a 2W HMGB1

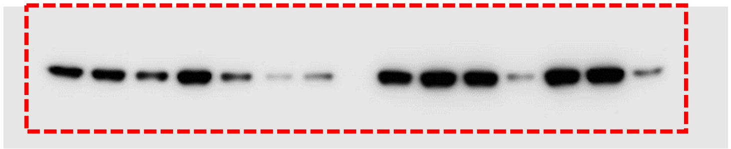

Fig. 5 a 4W HMGB1

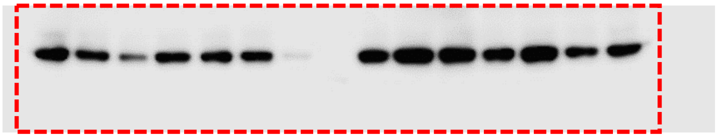

Fig. 5 a 6W HMGB1

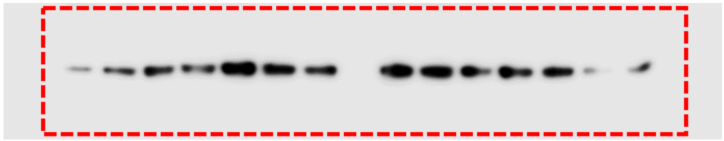

Fig. 5 d Atrogin1

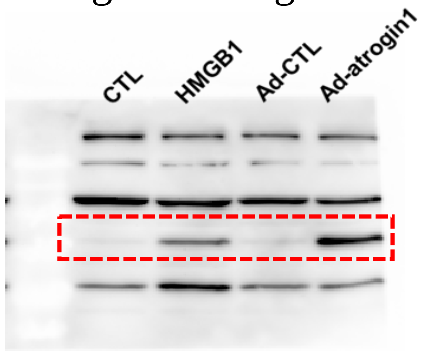

Fig. 5 d Ubiquitin

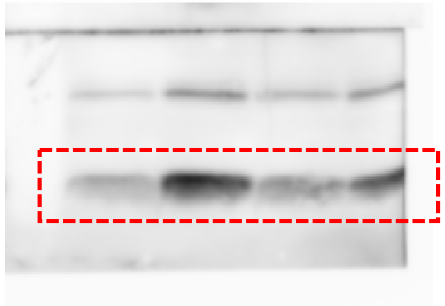

Fig. 5 d AQP4

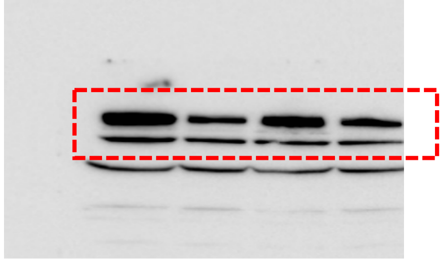

Fig. 5 d α-tubulin

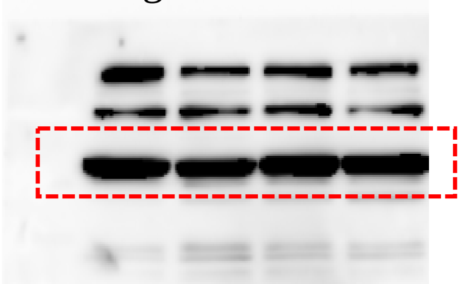

Fig. 5 e Ub-AQP4

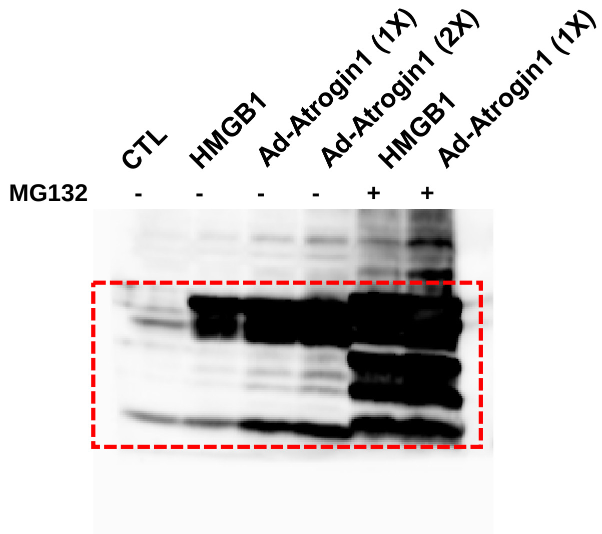

Fig. 5 e Atrogin1

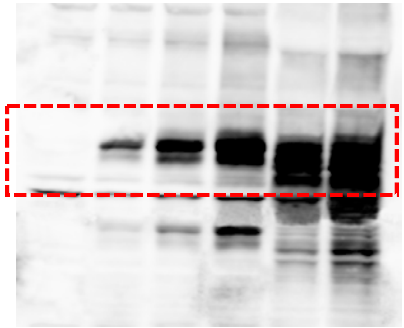

Fig. 5 e α-tubulin

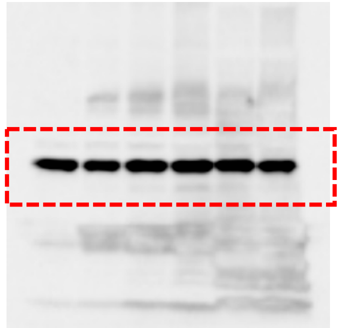

Figure S4

Fig. S4 AQP4

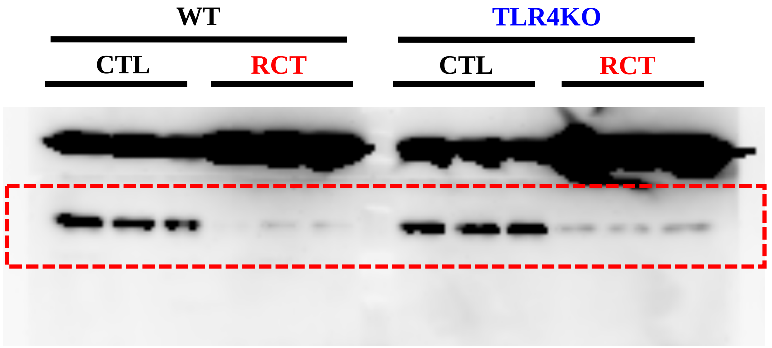

Fig. S4 α-tubulin

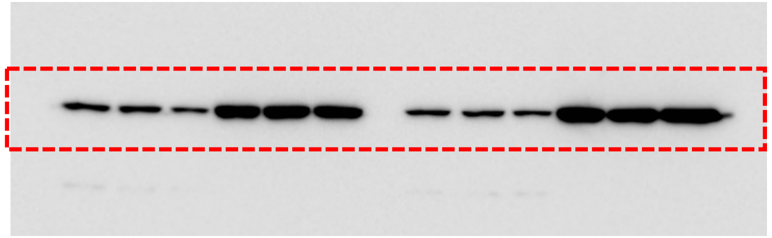

Supplement: Supplementary file 1 — Supplementary Information. [file 41598_2020_71167_MOESM1_ESM.pdf]
